# Mutations in sorghum *SBEIIb* and *SSIIa* affect alkali spreading value, starch composition, thermal properties and flour viscosity

**DOI:** 10.1007/s00122-019-03430-0

**Published:** 2019-10-17

**Authors:** Stefanie Griebel, Richard P. Westerman, Adedayo Adeyanju, Charles Addo-Quaye, Bruce A. Craig, Clifford F. Weil, Suzanne M. Cunningham, Bhavesh Patel, Osvaldo H. Campanella, Mitchell R. Tuinstra

**Affiliations:** 1 Department of Agronomy, Purdue University, Lilly Hall of Life Sciences, 915 W State Street, West Lafayette, IN 47907 USA; 2 College of Agriculture Administration, Purdue University, West Lafayette, IN 47907 USA; 3 Division Natural Sciences and Mathematics, Lewis-Clark State College, Lewiston, ID 83501 USA; 4 Department of Statistics, Purdue University, West Lafayette, IN 47907 USA; 5 Whistler Carbohydrate Research Center, Purdue University, West Lafayette, IN 47907 USA; 6 Department of Food Science and Technology, The Ohio State University, Columbus, OH 43210-1007 USA

## Abstract

**Key message:**

Seven novel alleles of *SBEIIb* and one allele of *SSIIa* co-segregated with the ASV phenotype and contributed to distinct starch quality traits important for food-processing applications.

**Abstract:**

Sorghum is an important food crop for millions of people in Africa and Asia. Whole-genome re-sequencing of sorghum EMS mutants exhibiting an alkali spreading value (ASV) phenotype revealed candidate SNPs in *Sobic.004G163700* and *Sobic.010G093400*. Comparative genomics identified *Sobic.010G093400* as a *starch synthase IIa* and *Sobic.004G163700* as a *starch branching enzyme IIb*. Segregation analyses showed that mutations in *Sobic.010G093400* or *Sobic.004G163700* co-segregated with the ASV phenotype. Mutants in *SSIIa* exhibited no change in amylose content but expressed lower final viscosity and lower starch gelatinization temperature (GT) than starches from non-mutant plants. The *sbeIIb* mutants exhibited significantly higher amylose levels and starch GT and lower viscosity compared to non-mutant starches and *ssIIa* mutants. Mutations in *SBEIIb* had a dosage-dependent effect on amylose content. Double mutants of *sbeIIb* and *ssIIa* resembled their *sbeIIb* parent in amylose content, starch thermal properties and viscosity profiles. These variants will provide opportunities to produce sorghum varieties with modified starch end-use qualities important for the beer brewing and baking industries and specialty foods for humans with diabetes.

**Electronic supplementary material:**

The online version of this article (10.1007/s00122-019-03430-0) contains supplementary material, which is available to authorized users.

## Introduction

Sorghum [*Sorghum bicolor* (L.) Moench] is important in drought-prone environments (Food and Agriculture Organization of the United Nations 2019a, b; Kimber 2000). It is a staple crop in Africa and Asia and is utilized to produce traditional foods such as stiff porridges and beverages (House et al. 2000). Indigenous to Africa, sorghum flour is less expensive than other imported flours (Taylor and Dewar 2000); thus, it has a great economic value for smallholder farmers.

Starch is a major component of sorghum flour and provides unique texture and nutritional value to foods (BeMiller 2014; BeMiller and Huber 2008; Biliaderis 2009). The two major starch fractions, amylose and amylopectin and their ratio, influence starch functionality (BeMiller and Huber 2008; Jane 2009; Tetlow et al. 2004), where amorphous (α-1,6 branch points) and crystalline (linear α-1,4 glucan chains) regions play important roles (Tetlow and Emes 2014). Starch compositions of 70–80% amylopectin and 15–30% amylose are reported in normal starches of maize, rice wheat and sorghum (Jane 2009; Biliaderis 2009; Shannon et al. 2009; BeMiller and Huber 2008; Waniska and Rooney 2000; Sang et al. 2008). Mutant starches such as waxy can have up to 100% amylopectin, and high amylose starches can have more than 50% apparent amylose content (Jane 2009; BeMiller and Huber 2008). Both polymers and their ratio can impact characteristics such as paste viscosity, gel consistency, thickening power and starch GT (BeMiller and Huber 2008; Bouvier and Campanella 2014; Shannon et al. 2009). The melting of the crystalline regions, mainly formed by amylopectin, is critical for starch granule swelling and starch gelatinization (BeMiller and Huber 2008; Bouvier and Campanella 2014). Some of the granule physio-chemical and structural characteristics are directly related to granule swelling and starch pasting properties (Desam et al. 2018) and are positively correlated with the peak viscosity (“the maximum swelling capacity of the starch granules”) (Bouvier and Campanella 2014).

Amylose levels in sorghum are positively correlated with final viscosity and setback (increase in viscosity after breakdown) and negatively with breakdown (Hill et al. 2012) and peak viscosity (Beta et al. 2000; Hill et al. 2012). The breakdown is a loss of viscosity of a starch paste and is genotype dependent (Bouvier and Campanella 2014). The viscosity breakdown can be described as the time range after peak viscosity occurred, when the starch granules fall apart due to shear forces while the temperature remains constant (Bouvier and Campanella 2014). The final viscosity develops over time during a temperature reduction in the sample, which is related to the development of starch gel viscosity (Bouvier and Campanella 2014) and consistency. Beta et al. (2000) reported a positive correlation between amylose content and peak starch GT (*T*_p_) in sorghum (Beta et al. 2000). Thus, end-product quality depends on the functionality of starches, which is influenced by starch characteristics and starch GT (BeMiller and Huber 2008; Biliaderis 2009). Sorghum beer is one example of an important end-product. The high starch GT of sorghum requires higher mashing temperatures, which result in low levels of fermentable sugars (Taylor and Dewar 2000; Taylor 1992). It is therefore desired to develop sorghum genotypes with lower starch GT to increase fermentable sugar output and thus beer quality. Food products containing resistant starches are important end-products consumed by humans with diabetes (Englyst et al. 2007). Resistant starches are correlated with high amylose content (Jane 2009; Tetlow and Emes 2014; Li et al. 2008) and exhibit reduced absorption in humans and demonstrate lower glycaemic index values (Englyst et al. 2007). *Amylose extender* (*ae*) is a mutant starch type resulting in high amylose starches in maize (Li et al. 2008; Liu et al. 2012a; Nakamura 2015b; Shannon et al. 2009; Tetlow and Emes 2014) and rice (Nakamura 2015b; Nishi et al. 2001; Tetlow and Emes 2014). The high amylose and branch chain length distribution are key influencers of starch digestibility (Tetlow and Emes 2014). The production of resistant starches in sorghum is poorly understood (Griebel et al. 2019).

The relative amounts and structural characteristics of amylose and amylopectin are under genetic control (Tetlow et al. 2004). The starch-synthesis pathway (SSP) is a conserved pathway across cereal species (Tetlow et al. 2004). Located in the endosperm of the seed, adenosine 5’ diphosphate glucose pyrophosphorylase (AGPase), granule bound starch synthases (GBSSs), starch synthases (SSs), starch branching enzymes (SBEs) and two types of debranching enzymes, isoamylases and pullulanases, are the key groups of enzymes involved in starch biosynthesis (Tetlow et al. 2004). The different enzymes vary in their catalytic activities (Guan and Preiss 1993; Nakamura 2015a; Tetlow et al. 2004; Tetlow and Emes 2014). Hill et al. (2012) reported that mutations in *SSIIa* and *SBEIIb* result in increased amylose values in sorghum. *Amylose extender* is regulated by *SBEIIb* (Jane 2009; Nakamura 2015b; Nishi et al. 2001; Tetlow and Emes 2014). The *Su2* gene codes for SSIIa in corn (Liu et al. 2012b; Zhang et al. 2004) and contribute to low starch GT (Liu et al. 2012b; Preiss 2009; Shannon et al. 2009; Zhang et al. 2004). High amylose in sorghum was associated with higher starch GT (Hill et al. 2012). The starch GT phenotype in rice is reported to be controlled by the SSSII-3 (ALK) gene encoding a starch synthase II (Gao et al. 2011), also controlling the ASV phenotype (Gao et al. 2011; Tian et al. 2009). In sorghum, the genes controlling the ASV phenotype and how ASV relates to starch characteristics is poorly understood.

A rapid assay to measure alkali spreading value (ASV) is commonly used in rice breeding to select for genotypes with varying starch GT (Little et al. 1958). The ASV phenotype is negatively correlated with starch GT in rice (Bhattacharya 1979; Bhattacharya and Sowbhagya 1972; Juliano et al. 1964; Mariotti et al. 2010; Tan and Corke 2002). Griebel et al. (2019) adapted the ASV test used in rice with a scale of 0–7 (Little et al. 1958) into a binary scale for use in sorghum to identify genotypes with modified starch properties. Mutants with an ASV+ phenotype exhibited lower to higher starch GTs compared to BTx623; thus, providing new opportunities to develop sorghum varieties with modified starch GT (Griebel et al. 2019). The ASV assay is of great benefit to preselect for varying starch GT genotypes but cannot replace DSC to differentiate lower and higher starch GT (Griebel et al. 2019). So far, it is poorly understood how the ASV phenotype of sorghum relates to different starch characteristics such as amylose content, resistant starch and paste viscosity profiles.

Given reports in rice, the initial hypothesis was that a *SSIIa* gene, involved in starch biosynthesis, controls the ASV phenotype in sorghum seed endosperm. However, many genes and enzymes are known to be involved in starch biosynthesis and processing and these genes may also contribute to variation in ASV. In this study, sorghum mutants (EMS ethyl methane sulfonate) exhibiting an ASV+ phenotype were used to identify the genes controlling the alkali spreading phenotype of sorghum based on whole-genome re-sequencing and genetic segregation analyses. Genotypes with single or double mutations were created to assess starch properties including amylose content, starch GT and paste viscosity profiles. Grain samples produced from these mutants were also characterized for variation in starch quality and related food-processing parameters. Genes, alleles and SNP markers for use in breeding sorghum varieties with varying starch GT and composition are reported.

## Materials and methods

### Plant material

A sorghum EMS population was developed using BTx623 (Krothapalli et al. 2013; Addo-Quaye et al. 2018). The ASV test was used in a forward genetic screen to identify *Sorghum bicolor* EMS (SbEMS) mutants with altered starch phenotypes (Griebel et al. 2019). Seed of nine SbEMS mutant lines exhibiting altered ASV phenotype were produced in Puerto Rico during the 2016–2017 production season (PR16/17). These lines and controls BTx623, Macia and Sepon82 were also grown in West Lafayette 2017 (WL17) and 2018 (WL18). The trials were grown in West Lafayette under rainfed conditions with long days. The trials in Puerto Rico were grown under short day conditions with irrigation. Two biological replicates per genotype and year except three replicates of BTx623 in WL18 were tested for ASV, amylose content and paste viscosity profiles.

To conduct an allele dosage test, A-lines for each mutant were developed by crossing each mutant line as a pollen donor to ATx623 followed by backcrossing to the *F*_1_ over several generations. The mutant B-lines were used as the recurrent parents in each generation. The mutant B-lines are registered as genetic stocks in the USDA National Plant Germplasm System at the National Laboratory for Genetic Resources Preservation (PI 690479 SbEMS 2703; PI 690480 SbEMS 2773, PI 690481 SbEMS 3194, PI 690482 SbEMS 3218, PI 690483 SbEMS 3403, PI 690484 SbEMS 3568, PI 690485 SbEMS 3920, PI 690486 SbEMS 4308, PI 690487 SbEMS 5890).

Double mutants were created by hand emasculating and crossing selected EMS mutants in 2017 (SbEMS 3194 × SbEMS 4308; SbEMS 2703 × SbEMS 4308). The harvested *F*_1_ seeds were grown in the greenhouse and genotyped for both parental alleles. The verified *F*_1_ plants were self-pollinated, and the *F*_2_ populations were grown in a greenhouse and genotyped to identify homozygous double mutant *F*_2_ plants. The homozygous double mutants were transplanted in the field in 2018, self-pollinated and harvested as single panicles. The parental genotypes were also grown in the field in 2018. The seeds of double mutant lines and two biological replicates for each of the parent lines were subjected to further phenotypic analyses.

### DNA preparation

DNA was extracted from the SbEMS mutants exhibiting an ASV+ phenotype (Griebel et al. 2019) using a modified CTAB extraction protocol (Murray and Thompson 1980). Tissue samples were ground in extraction buffer solution (1.2 M NaCl, 100 mM Tris pH 8, 20 mM EDTA pH8, 2% CTAB, 0.1% BME) followed by extraction with chloroform (24 parts chloroform, 1 part isoamyl alcohol). The aqueous layer was transferred to a new Eppendorf tube and diluted with 1070µL dilution buffer (100 mM Tris pH 8, 20 mM EDTA pH 8, 2% CTAB) followed by precipitation with 530µL wash buffer (3 parts TE buffer, 7 parts ethanol). The samples were incubated for 15 min at room temperature, centrifuged at 12,000 (3220 × *g*) for 10 min, and the pellet was re-suspended in 53 µL high-salt TE [1 M NaCl, 10 mM Tris pH 8, 2 mM EDTA pH 8, RNaseA (50 µg/mL)]. The samples were incubated at 60 °C for 15 min and then diluted with 212 µL H_2_O. DNA was precipitated with 530 µL pure EtOH at − 80 °C for 30 min, centrifuged (3220 × *g*) for 10 min, and the pellet was washed with 70% ethanol. The pellets were re-suspended in TE buffer (10 mM Tris pH 8, 1 mM EDTA pH 8).

The fast DNA extraction method described by Xin et al. (2003) was used for DNA extractions for co-segregation analyses.

### Whole-genome re-sequencing and analyses

Whole-genome re-sequencing data for all the sorghum samples were generated at the Purdue Genomic Core Facility. One sorghum mutant, SbEMS 3218, had undergone next-generation sequencing as described by Addo-Quaye et al. (2018), and SNP data were reported at the Functional Gene Discovery Platform for Sorghum (https://www.purdue.edu/sorghumgenomics/). The other eight EMS mutants SbEMS 2703, SbEMS 2773, SbEMS 3194, SbEMS 3403, SbEMS3568, SbEMS 3920, SbEMS4308 and SbEMS 5890 were processed as described below. The library construction was conducted using the TruSeq^®^ DNA PCR-Free Sample Preparation Guide from Illumina. The samples SbEMS 2773, SbEMS 3194, SbEMS 3568 and SbEMS 5890 were PCR amplified as the final library concentration was too low after the PCR-free protocol step. The samples were sequenced using Illumina HiSeq 2500 with 100 bp paired-end reads. The filtered NGS read files (R1 and R2) obtained from the Purdue Genomics Core were cleaned of contaminants by aligning the R1 and R2 files to the indexed Phix174 genome using *Bowtie 2* short reads aligner (Langmead and Salzberg 2012). Next, NGS reads with origins in the sorghum mitochondrial genome were filtered by using *Bowtie 2* to detect alignment to the mitochondrial genome NC 008360.1 downloaded and freely available from the National Center for Biotechnology Information (NCBI) sequence read archive at https://www.ncbi.nlm.nih.gov/sra.

A reference genome-based read mapping approach was used for SNP calling and quality filtering. The *Sorghum bicolor* reference genome BTx623 (Paterson et al. 2009) assembly and annotation sequences (version 3.0; *Sbicolor_313_v3.1*) were downloaded from *Phytozome* database version 11 (Goodstein et al. 2012). The reference genome assembly sequences were indexed using Bowtie2 *build*. The filtered NGS paired-end reads were aligned to the indexed sorghum reference genome using *Bowtie 2* (Langmead and Salzberg 2012). The reference genome sequences were indexed separately using the *faidx* and *CreateSequenceDictionary* programs in the SAMtools alignment manipulation software (Li et al. 2009) and the GATK genome analysis toolkit packages (McKenna et al. 2010; DePristo et al. 2011), respectively. The tool GATK was used for SNP calling (McKenna et al. 2010; DePristo et al. 2011). The SAM-formatted output files containing the aligned sorghum NGS reads were sorted and indexed using the *sort* and *index* programs in the *SAMtools* software package. NGS duplicate reads were tagged using the *MarkDuplicates* tool in the *Picard Tools* package (Picard Toolkit 2016). Variant detection was performed in the sorted BAM files using the *GATK HaplotypeCaller* tool (DePristo et al. 2011; McKenna et al. 2010). The SNP data were submitted as.vcf files to the European Variation Archive (EVA) and can be found under the EVA project PRJEB33472 named “Sorghum EMS mutants exhibit ASV and special starch quality traits.” The SNP data are also made available via PURR—the Purdue University Research Repository, and can be found at https://purr.purdue.edu/publications/3238/1.

### Functional annotations of SNP variation

The effects of identified SNPs on the sorghum gene function were found using *snpEff* variant effect annotation tool (Cingolani et al. 2012). The default *snpEff* configuration file was updated with a new database name for the sorghum genome. The *Sorghum bicolor* reference genome sequences (FASTA-formatted file) and the genome annotation information (*gff3* file) obtained from *Phytozome* database version 11 (Goodstein et al. 2012) were used to create a new database using *snpEff build*-*gff3* command. Then .vcf files that contain the GATK Haplotype Caller variants were used as input files for the next step. In this next step, a snpEff-annotated variant call *.vcf* output file containing the variant annotations was created.

### Designation of starch-synthesis genes (candidate gene approach)

A list of candidate genes involved in sorghum starch biosynthesis in the amyloplast was made to check for SNPs in all the genes associated with starch biosynthesis. The majority of genes involved in the amyloplast were obtained from Campbell et al. (2016). The genes *Sobic.007G101500* and *Sobic.009G127500* were also added to this list based on recent studies (Goodstein et al. 2012, database version 11).

### Comparative genomics: multiple sequence alignment

The protein sequences from *Sobic.004G163700* and *Sobic.010G093400* were downloaded from *Phytozome.* The sequence was blasted against all green plants, viridiplantae, in NCBI using tBLASTn (Altschul et al. 1990; Camacho et al. 2009). More and less related species were selected to be able to see whether the detected SNPs are in conserved protein regions across more related species or even across all species. The protein sequences were then downloaded in fasta format. Multiple sequence alignment was conducted using Clustal Omega (Chojnacki et al. 2017), and alignment results were imported into the ClustalW2 package Simple Phylogeny for calculation of distances by the unweighted pair group method algorithm (UPGMA) method. The alignment and clustering packages are freely available from https://www.ebi.ac.uk/Tools/.

### Primerdesign, PCR and SNP genotyping (CAPS, dCAPS, tetra-primer)

CAPS (Cleaved Amplified Polymorphic Sequences) markers were created for the mutant alleles *sbeIIb*-*2773*-*2, sbeIIb*-*5890*-*7* and *sbeIIb*-*3568*-*6*. The sequence around the SNP was obtained from *Phytozome*. The software dCAPS Finder 2.0 (Neff et al. 2002) is freely available at http://helix.wustl.edu/dcaps/ and was used with 0 mismatches to find restriction enzymes cutting both forward and reverse strand and only the mutant or wild-type, not both. NCBI Primer-BLAST (Altschul et al. 1990; Camacho et al. 2009) was used to create primers and is freely available from https://www.ncbi.nlm.nih.gov/tools/.

dCAPS (Derived Cleaved Amplified Polymorphic Sequences) were created for the mutant allele *sbeIIb*-*3194*-*3*. The sequence around the SNP was obtained from *Phytozome*. The dCAPS Finder 2.0 (Neff et al. 2002) was used to create dCAPS with maximum of 2 mismatches.

Tetra-primer for ARMS-PCR were created for the mutant alleles *ssIIa*-*4308*-*2, sbeIIb*-*2703*-*1, sbeIIb*-*3403*-*5* and *sbeIIb*-*3218*-*4*. The sequence around the SNP was obtained from the reference genome. The software PRIMER1: primer design for tetra-primer ARMS-PCR, freely available at http://primer1.soton.ac.uk/primer1.html, was used to create tetra-primer (Collins and Ke 2012; Ye et al. 2001).

Primers used for PCR amplification are described in Table 1. The PCR reactions were adjusted to primer-specific annealing temperatures for each mutant. The ratio and amount of primers used were adjusted for tetra-primer types. Polyvinylpyrrolidone (PVP) 20% and bovine serum albumin (BSA) were added to improve PCR amplification of DNA from fast and dirty extractions (Xin et al. 2003).

**Table 1:** PCR primers, primer sequences and restriction enzymes used for the genotyping of sorghum EMS mutants

PCR primer ID/type	Gene/SNP position/allele	Primer sequence	Temp. (°C)	Enzyme cut	Product size
2703-3_ S12_51294121 Tetra-primer	*Sobic.004G163700* 51294121 *sbeIIb*-*2703*-*1*	InF: TTGCATTGCCTGATCAAACTCGTA InR: CGGACTATCTTAGGTATCGTGGTAGGC OuF: GACATTACAAGAAGAATCCCCACCAA OuR: GTTTGGTCAATAATTGATCATTGTCGG	65.2	None	Product size T allele: 110 C allele: 165 outer primers: 224
2773_ 04_SNP_51302688 CAPS	*Sobic.004G163700* 51302688 *sbeIIb*-*2773*-*2*	F: CACGGTAAAGAGTACCTGCGA R: TGAGCATGAAGGAGGCTTGG	57.1	ApoI-HF cuts Mutant (M)	~ 78 (cut ~ 28, ~ 50 bp)
3194_ 04_SNP_51298115 dCAPS	*Sobic.004G163700* 51298115 *sbeIIb*-*3194*-*3*	F: ATCATGGAGGTCACACCATCAATGC R: TCCAATGCTAGATGGTGGCTTGAG	59.1	HpyCH4 V cuts M	~71 (cut ~ 26, ~ 45)
3218 S_TET Tetra-primer	*Sobic.004G163700* 51295327 *sbeIIb*-*3218*-*4*	InF: GTGTGTGCACAATATCACCCATCGTT InR: CAGGCAAAGTGATGAAGCTGGG OuF: ACCTTGTCCATCAACCAAAATGCA OuR: CGTGTGCGTTCACTTTGAGCTATG	61.0	None	Product size G allele: 150 A allele: 100 outer primers: 202
3403(1) Tetra-primer	*Sobic.004G163700* 51295906 *sbeIIb*-*3403*-*5*	InF: AGTTCAATCCATTTGTCAGCCACATCT InR: GTAGGTTTTGACTATCGGATGCACCTG OuF:CAGCCCATGCAATTAAACATTAGTGTATG OuR: CCTCCACATCATTGGCTTACATAAACC	60.0	None	Product size A allele: 201 G allele: 182 outer primers: 329
3568_ 04_SNP_51292780 CAPS	*Sobic.004G163700* 51292780 *sbeIIb*-*3568*-*6*	F: ACATCTGTGTACCAAAGGCGAT R: CAGGATCCATCACGCAGCA	57.2	HpyCH4III cuts WT	~79 (cut ~ 37, ~ 42)
4308_ S13_8302675 Tetra-primer	*Sobic.010G093400* 8302675 *ssIIa*-*4308*-*2*	InF: TTGTTCTGCAAGGTTGCTGGTA InR: TGACAGTATAGTTCAGGGGGATACATC OuF: TGATGCACCTCTCTTCCGG OuR: TTCGCTAGTGCAAAAGTTGATCC	61.6	None	Product size A allele: 181 G allele: 224 two outer primers: 356
5890_ 04_SNP_51301340 CAPS	*Sobic.004G163700* 51301340 *sbeIIb*-*5890*-*7*	F: TGCTAGCCCAAAGTAGGAACAA R: CAGGCTCCAGGAGAAATACCA	56.4	MboI cut WT 1x, M 2x	~121 (cut: *W*: ~ 78, 43; *M*: ~ 67, 11, 43)

Each PCR product from CAPS and dCAPS was digested with enzymes from New England Biolabs under recommended conditions. The allele *sbeIIb*-*3568*-*6* (3568_04_SNP_51292780) was digested with HpyCH4III, *sbeIIb*-*2773*-*2* (2773_04_SNP_51302688) with ApoI-HF, *sbeIIb*-*5890*-*7* (5890_04_SNP_51301340) with MboI and *sbeIIb*-*3194*-*3* (3194_04_SNP_51298115) with CviRI=HpyCH4 V. All samples from enzyme digest or tetra-primers were examined in 2.5–3% TAE high-resolution agarose gels, depending on the fragment size.

### Co-segregation analyses

The SbEMS mutants were crossed to BTx623 (except SbEMS3920 was un-successful in crossing). *F*_1_ plants were selected and self-pollinated to create bi-parental *F*_2_ populations. Panicles of *F*_2_ plants were self-pollinated, and *F*_2:3_ seeds were screened for ASV with 32 seeds per panicle and in rare cases less if limited by seed number. The number of panicles per population screened varied from 30 for SbEMS3403 to 112 for SbEMS2773. *F*_3_ plants of those *F*_2:3_ panicles were grown on a bench containing sand, and leaf tissues were sampled and genotyped. Each panicles’ ASV phenotype and the corresponding SNP genotype (*M* = mutant, *H* = heterozygous, *W* = wild-type) was determined. A Chi-square test was conducted to determine goodness of fit for the 3:1 phenotypic ratio as well as the 1:2:1 genotypic ratio.

### Allele dosage test

An allele dosage test was conducted to determine whether the mutant alleles influenced amylose content in an allele-dependent manner. The A-lines and B-lines for each mutant were intercrossed to produce the hybrids for testing inheritance in the triploid endosperm. To obtain zero doses of the mutant allele, BTx623 was used as pollen donator and crossed to ATx623. A single dosage of the mutant allele was created by crossing the mutants onto ATx623. Two dosages of the mutant allele were created by crossing BTx623 onto the A-line (or hand emasculated panicle) of each EMS mutant. Three doses were created by self-pollinating the B-line (West Lafayette 2017) or by crossing the B-line to the corresponding A-line (West Lafayette 2018). The hybrid seeds were analyzed for ASV and amylose content. The experiment was set up to obtain two biological replicates per genotype and allele dosage, but depended on crossing success and seed set in the field. In 2017, the A-line for SbEMS2703 was not available so hybrids were made by hand emasculation. The SbEMS3920 mutant was not included as no co-segregation data were available at that point.

### Phenotypic analyses

The alkali spreading test was conducted as described in Griebel et al. (2019) with 1.8% KOH for 24 h and scored binary as ASV+ or ASV− (wild-type). Thirty-two seeds were randomly chosen and tested. In rare cases, fewer seeds were tested if seed quantity was limited. The randomly chosen seeds for the allele dosage test were cut horizontally so that only the triploid endosperm was evaluated for ASV with 32 seeds per panicle.

The flour preparation was conducted as follows. The seed samples from PR16/17, WL17 and WL18 described above were milled into a fine flour using a ball mill (Retsch, Haan, Germany) as described in Griebel et al. (2019).

The starch extraction and thermal properties were conducted as previously described from flour samples of double mutants and parents (Griebel et al. 2019; Benmoussa and Hamaker 2011). The flour samples of double mutants and parents were analyzed in duplicate for their starch thermal properties using differential scanning calorimetry (DSC) as described by Griebel et al. (2019). The starch thermal properties tested were onset (*T*_o_), peak (*T*_p_), conclusion (*T*_c_) starch GT, range of starch GT (*T*_c_ − *T*_o_) and enthalpy.

The paste viscosity analysis was conducted, and corresponding samples were adjusted based on moisture content. Moisture was determined using the standard oven method (Bradley Jr. 2014). Aluminum pans were oven-dried before use. For moisture determination, 1 g of flour sample was weighed on pre-weighed aluminum pans and dried over night at 105 °C. In rare cases, flour was limited and less than 1 g was sampled. The samples were placed in a desiccator after drying and before the final weight was recorded. The weights before and after drying were recorded, and moisture was calculated based on water loss. Paste viscosity analysis was performed on whole grain flour samples using the Rapid Visco Analyzer (RTE-100), model 4 (Serial Number 970835) from Newport Scientific, Australia. The RVA standard method 1 was used, and the results were analyzed using standard analysis 1. Water–flourdispersions of 11.86% were prepared (Bouvier and Campanella 2014). For the SbEMS mutants, two biological replicates from WL17 and WL18 were analyzed. Each biological replicate was sampled in duplicate. The double mutants from WL18 did not have enough flour sample, so in some cases the biological replicate was not analyzed in duplicate.

Amylose and amylopectin analyses were performed on whole grain flour samples by following the Megazyme K-AMYL kit manual. Each flour sample from PR16/17, WL17 and WL18 was analyzed in duplicate. The spectrophotometer (Spectronic Genesys 10 Bio, Thermo Electron Corporation, Model: 970S0008) was used at 510 nm.

### Statistical analyses

Statistical analyses were performed using SAS version 9.4. (The data analysis for this paper was generated using SAS software, Version 9.4 of the SAS System for Windows. Copyright © 2002–2012. SAS Institute Inc. SAS and all other SAS Institute Inc. product or service names are registered trademarks or trademarks of SAS Institute Inc., Cary, NC, USA.) The GLIMMIX procedure was used to perform a linear mixed model analysis of variance (ANOVA) with the Tukey–Kramer multiple comparison adjustment to compare starch GT, amylose and viscosity properties across genotypes and environments.

## Results

### Whole-genome re-sequencing of ASV mutants reveals SNPs in starch biosynthesis genes

**Fig. 1:**
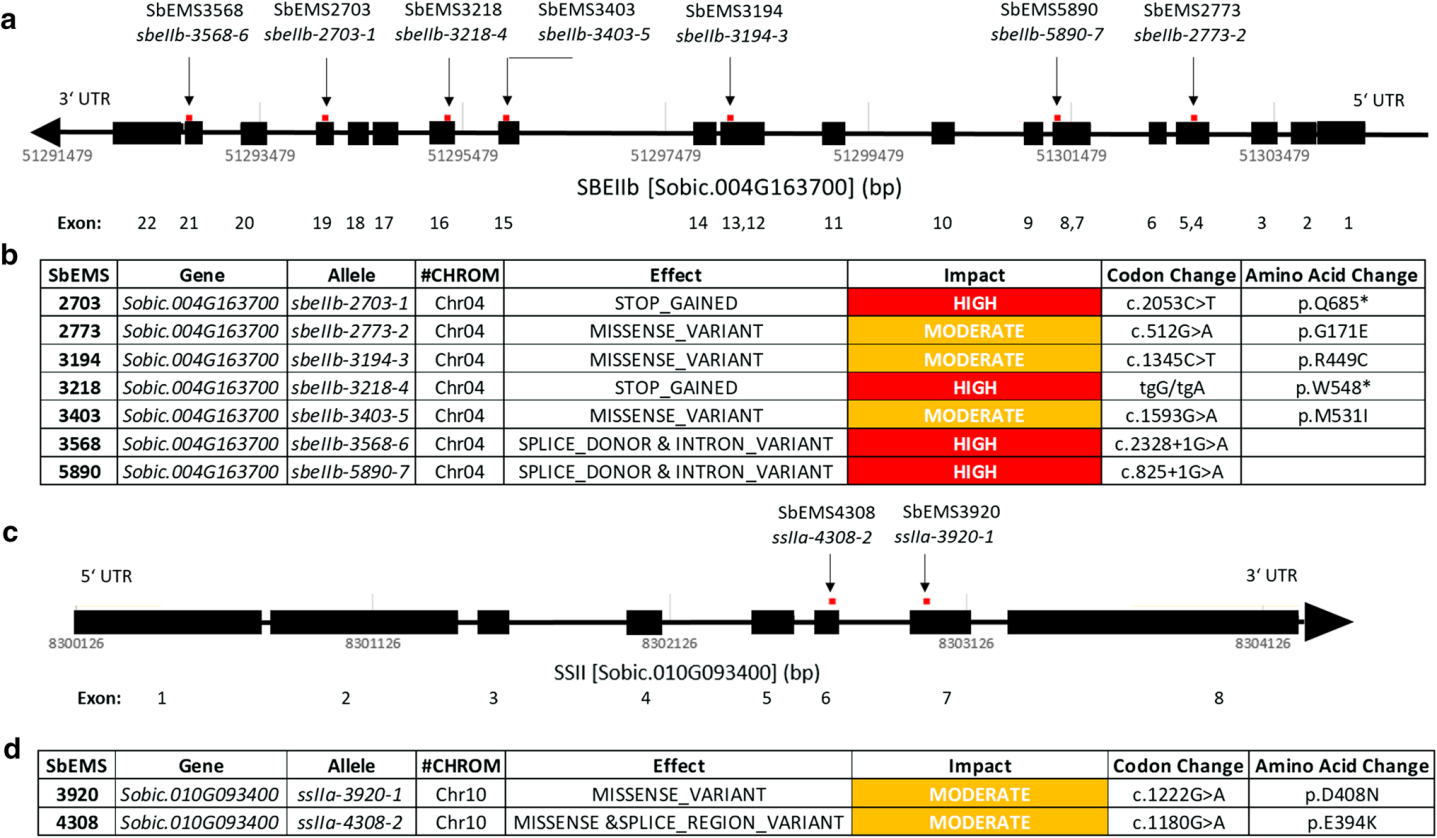
Mutations in *SBEIIb* and *SSIIa***a** mutants and positions of SNPs in *sbeIIb* (*Sobic.004G163700*), **b***SBEIIb* mutations, effects and impacts predicted by snpEff on transcription and translation, **c** mutants and positions of SNPs in *ssIIa* (*Sobic.010G093400*), and **d***ssIIa* mutations, effects and impacts predicted by snpEff on transcription and translation

Whole-genome re-sequencing studies characterizing the sorghum mutants described by Griebel et al. (2019) revealed homozygous SNPs in two (*Sobic.004G163700, Sobic.010G093400*) of 19 genes associated with starch biosynthesis (Fig. 1). SbEMS 2703, SbEMS 2773, SbEMS 3194, SbEMS 3218, SbEMS 3403, SbEMS 3568, SbEMS 5890 exhibited homozygous SNPs in *Sobic.004G163700,* described as a 1-4-alpha glucan starch branching enzyme II (Goodstein et al. 2012, database version 11; Campbell et al. 2016). The mutant alleles of this gene were described as *sbeIIb*-*2703*-*1, sbeIIb*-*2773*-*2, sbeIIb*-*3194*-*3, sbeIIb*-*3218*-*4, sbeIIb*-*3403*-*5, sbeIIb*-*3568*-*6 and sbeIIb*-*5890*-*7*, respectively. SbEMS 3920 and SbEMS 4308 exhibited homozygous SNPs in *Sobic.010G093400,* described as a starch synthase zSTSII-1 (Campbell et al. 2016). The mutant alleles of this gene were described as *ssIIa*-*3920*-*1 and ssIIa*-*4308*-*2*, respectively. The mutant SbEMS 3568 has a second homozygous SNP in the gene ADP (*Sobic.007G101500*), which was not further evaluated as it is an Intron-SNP and the ASV phenotype co-segregated with an exon-SNP in *Sobic.004G163700* for that mutant. Similarly, SbEMS 5890 has a second homozygous SNP in a starch synthase gene (*Sobic.010G093400*); predicted to be a synonymous variant and was, therefore, excluded from further consideration.

The snpEff tool (Cingolani et al. 2012) was used for functional SNP annotations to predict the effects of each SNP on transcription and translation of *Sobic.004G163700* and *Sobic.010G09340* (Fig. 1b, d). In *Sobic.004G163700*, the SNPs within the alleles *sbeIIb*-*3568*-*6* and *sbeIIb*-*5890*-*7* are predicted to alter splice donor sites and *sbeIIb*-*2703*-*1* and *sbeIIb*-*3218*-*4* created premature stop codons. Moderate protein effects in the form of missense variants (amino acid changes) are predicted for the SNPs in the alleles of *sbeIIb*-*2773*-*2*, *sbeIIb*-*3403*-*5* and *sbeIIb*-*3194*-*3*. In *Sobic.010G093400*, the SNP in the allele *ssIIa*-*3920*-*1* is predicted as a missense variant and the SNP in the allele *ssIIa*-*4308*-*2* is predicted as a missense and splice region variant.

### Comparative genomics identifies *SSIIa* and *SBEIIb*

**Fig. 2:**
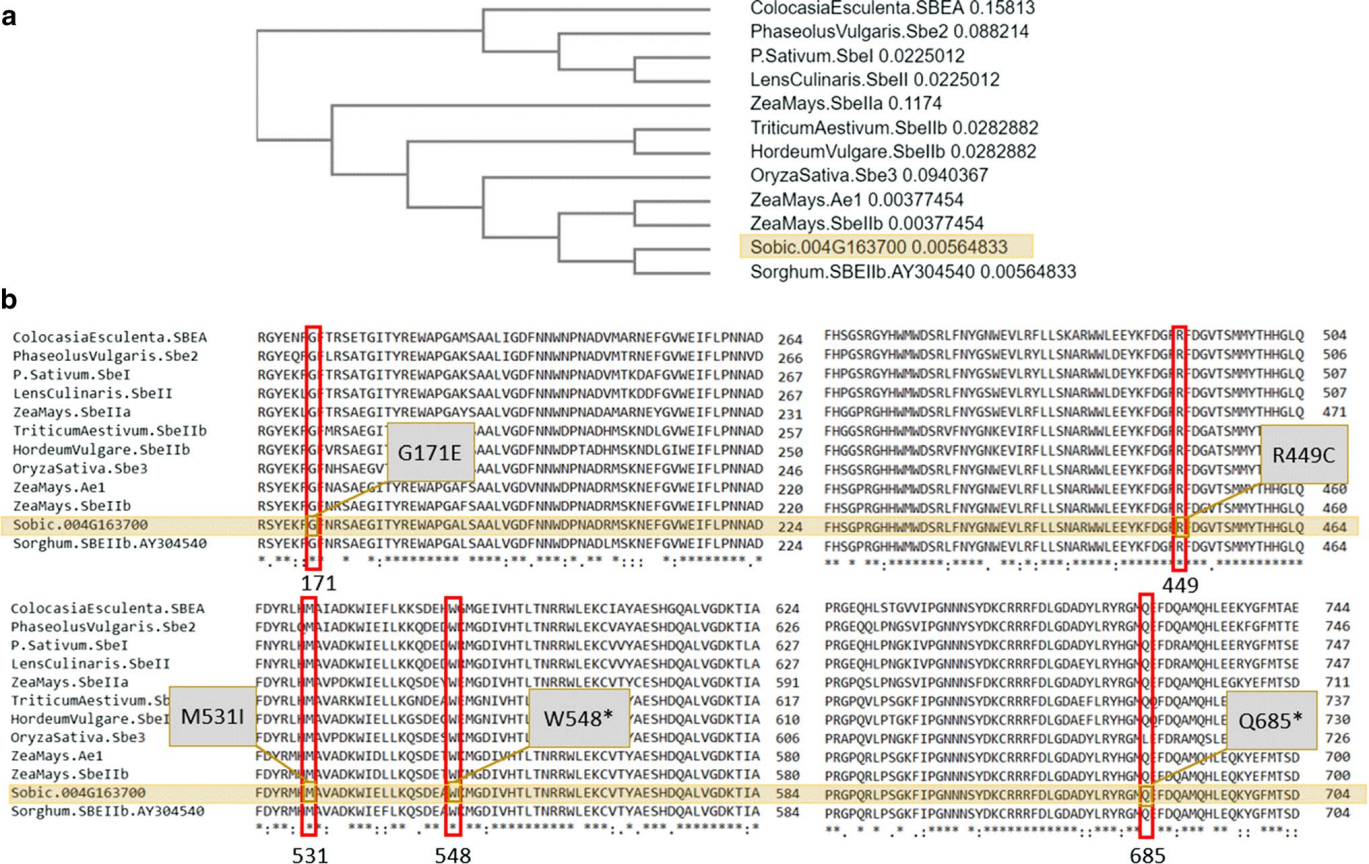
Multiple sequence alignment of protein sequences from different sources with sorghum starch branching enzyme: **a** phylogram using UPGMA and distance correction **b** SNPs in regions of the *Sobic.004G163700* protein compared to other species

Multiple sequence alignment and BLASTP (Altschul et al. 1990; Camacho et al. 2009) showed that *Sobic.004G163700* is likely the previously reported sorghum *SBEIIb* (AY304540) (Mutisya et al. 2003; De Alencar Figueiredo et al. 2008) as both show 98.9% sequence similarity (Goodstein et al. 2012, database version 12). Comparisons of the protein sequences and a phylogram (Chojnacki et al. 2017) of *Sobic.004G163700* showed that the new alleles are predicted to affect regions of the protein highly conserved across species like *Zea mays, Oryza sativa, Hordeum vulgare, Triticum aestivum, Lens culinaris, Pisum sativum, Phaseolus vulgaris* and *Colocasia esculenta* (Fig. 2). The similarity of the SBEIIb in sorghum, followed by maize, rice (sbe3), barley and wheat suggests that the identified gene is a starch branching enzyme of the isoform *SBEIIb*. This suggested that *Sobic.004G163700* encodes a *SBEIIb*.

**Fig. 3:**
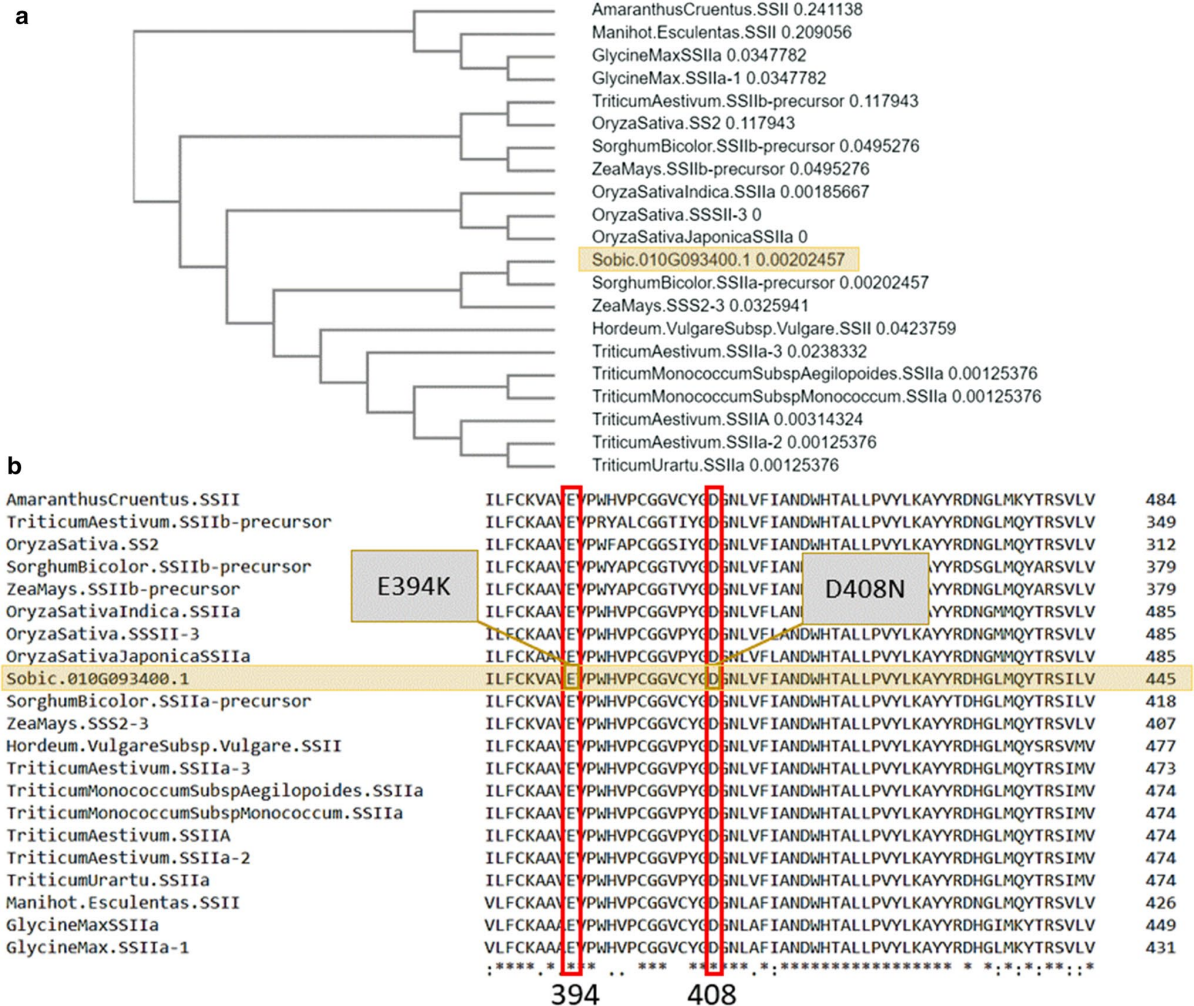
Multiple sequence alignment of protein sequences from different sources with sorghum starch synthase enzyme: **a** phylogram using UPGMA and distance correction **b** SNPs in protein regions of *Sobic.010G093400* compared to other species

Multiple sequence comparison of protein sequences (Chojnacki et al. 2017) similar to that encoded by *Sobic.010G093400* showed that the SNPs of *ssIIa*-*3920*-*1 and ssIIa*-*4308*-*2* are in regions highly conserved across species of *Glycine max, Triticum aestivum, T. monococcum* and *T. urartu, Hordeum vulgare, Zea mays, Oryza sativa* and *Amaranthus cruentus* (Fig. 3). The phylogenetic tree showed the sorghum *Sobic.010G93400* is most closely related to a sorghum SSIIa followed by SSS2-3 from maize and SSIIa from *Triticum*.

### ASV phenotype co-segregates with SNPs in *SSIIa* and *SBEIIb*

**Fig. 4:**
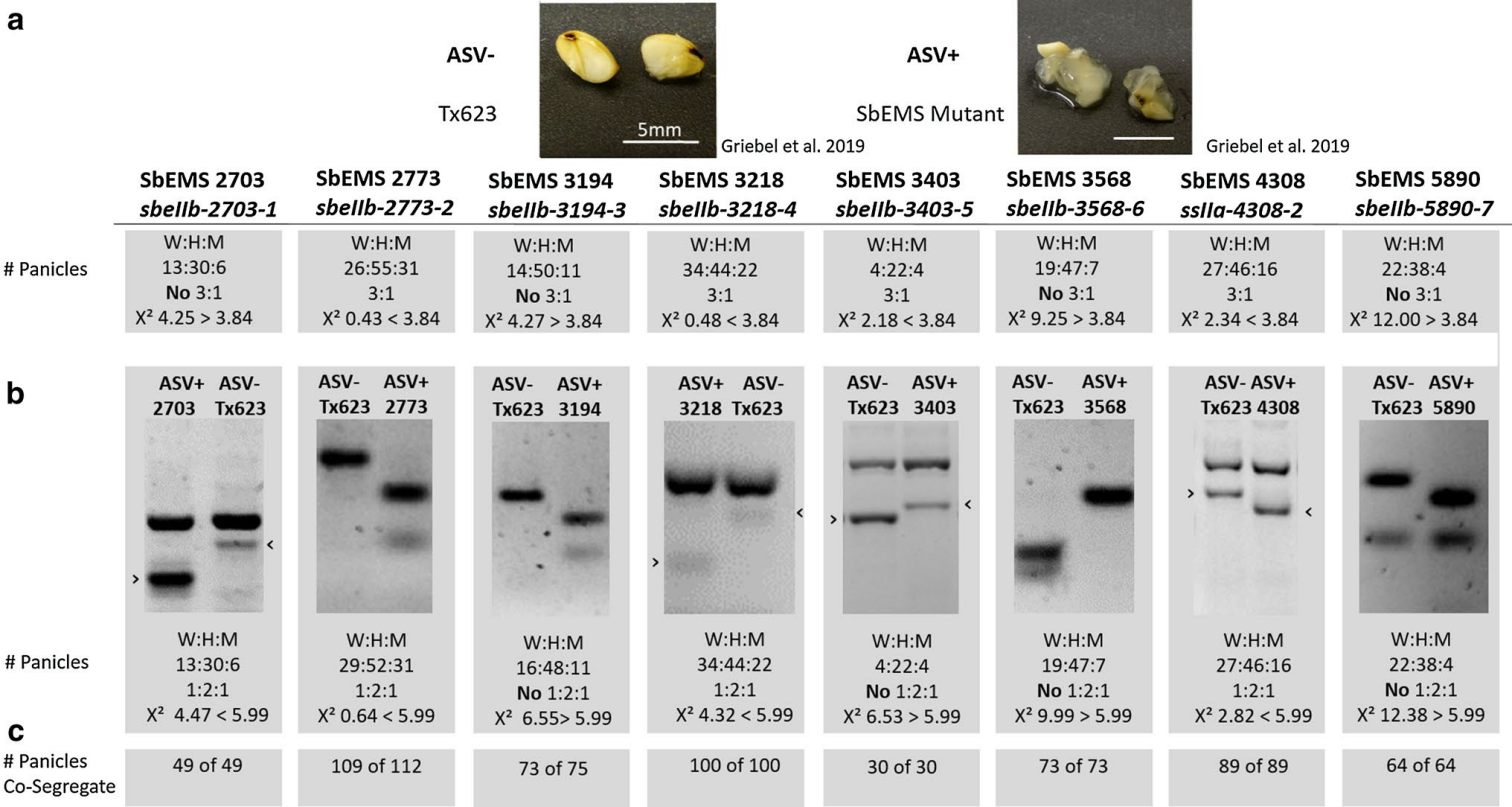
Co-segregation analyses—**a** of contrasting ASV phenotypes (1.8% KOH, 24 h), ASV segregation ratios for each SbEMS mutant, and statistical tests for segregation of ASV, **b** SNP markers for each SbEMS mutant, genotype segregation ratios and statistical tests for SNP segregation, and **c** co-segregation of SNP markers and ASV. Arrows (>) on gel pictures indicate the inner primers’ band of the tetra-primers used

Eight of the SbEMS mutants were crossed to BTx623 and evaluated in *F*_2:3_ populations for their ASV phenotype and associated SNP genotype (Fig. 4). Phenotypic evaluations and Chi-square tests provided evidence that the mutant alleles *sbeIIb*-*2773*-*2, sbeIIb*-*3218*-*4*, *sbeIIb*-*3403*-*5* and *ssIIa*-*4308*-*2* segregated for ASV in a 3:1 ratio (Fig. 4a). The 3:1 ratio did not fit (Chi-square *p* value) for the alleles *sbeIIb*-*2703*-*1, sbeIIb*-*3194*-*3, sbeIIb*-*3568*-*6* and *sbeIIb*-*5890*-*7*. Genotyping studies showed that the *sbeIIb*-*2703*-*1, sbeIIb*-*2773*-*2*, *sbeIIb*-*3218*-*4* and *ssIIa*-*4308*-*2* alleles segregated in a 1:2:1 genotypic ratio but *sbeIIb*-*3194*-*3*, *sbeIIb*-*3403*-*5*, *sbeIIb*-*3568*-*6* and *sbeIIb*-*5890*-*7* alleles exhibited distorted ratios (Fig. 4b). This may be due to low viability of the seeds for homozygous mutants. A larger sample size might have fit the 3:1 and 1:2:1 ratios better.

Linkage analyses demonstrated perfect co-segregation between the putative causal SNP markers and the ASV mutant phenotype in bi-parental populations of BTx623 with SbEMS 2703, SbEMS 3218, SbEMS 3403, SbEMS 3568, SbEMS 4308 and SbEMS 5890 (Fig. 4). Genetic analyses in populations developed using SbEMS 2773 and SbEMS 3194 also exhibited strong linkage between the SNP markers and the ASV phenotype. But three progenies for the variant *sbeIIb*-*2773*-*2* and two progenies for *sbeIIb*-*3194*-*3* exhibited a panicle with a heterogenous ASV phenotype but leaf tissue of a wild-type genotype.

### SSIIa and SBEIIb mutations result in different starch GT patterns

The mutants SbEMS 3920 and SbEMS 4308 showed significantly lower starch GT compared to the controls and the other mutants (Griebel et al. 2019). In the current study, it was determined that these genotypes have mutations in *Sobic.010G093400* as represented by *ssIIa*-*3920*-*1* and *ssIIa*-*4308*-*2*. SbEMS 2703, SbEMS 2773, SbEMS 3194, SbEMS 3218, SbEMS 3403, SbEMS 3568 and SbEMS 5890 exhibited the highest starch GT values, approximately 5 °C higher *T*_p_ than the control samples (Griebel et al. 2019). These genotypes were identified as having mutations in *Sobic.004G163700* as represented by *sbeIIb*-*2703*-*1, sbeIIb*-*2773*-*2, sbeIIb*-*3194*-*3, sbeIIb*-*3218*-*4, sbeIIb*-*3403*-*5, sbeIIb*-*3568*-*6* and *sbeIIb*-*5890*-*7.*

### Mutations in sorghum *SBEIIb* result in increased amylose values

Comparative studies showed that *Sobic.004G163700* is similar to SBEIIb (Mutisya et al. 2003) in sorghum and closely related to *Zea mays* amylose extender gene *Ae1*; hence, it was hypothesized that mutants with SNP mutations in this gene could have increased amylose values in comparison with controls and *SSIIa* variants (Table 2). There was no significant genotype by environment interaction (Table S1) observed across three seasons (Puerto Rico 2016/2017, West Lafayette 2017 and 2018), so amylose values are presented for each environment and in a combined analysis. Macia was not evaluated in Puerto Rico 2016/17 but exhibited amylose values similar to the other controls in the other environments.

**Table 2:** Amylose values for *Sorghum bicolor* EMS mutants and controls from Puerto Rico 2016/2017 (PR16/17), West Lafayette 2017 (WL17) and West Lafayette 2018 (WL18)

Pedigree	Allele	PR 16/17	WL 2017	WL 2018	Combined	PR 16/17	WL 2017	WL 2018
		Amylose%–LSMeans				Rank		
EMS2703	*sbeIIb*-*2703*-*1*	43.54 C	43.60 BC	38.14 BC	41.76 D	2	2	3
EMS2773	*sbeIIb*-*2773*-*2*	38.16 C	37.89 BC	35.79 BC	37.28 C	5	6	6
EMS3194	*sbeIIb*-*3194*-*3*	44.02 C	48.15 C	45.10 C	45.76 E	1	1	1
EMS3218	*sbeIIb*-*3218*-*4*	41.40 C	40.54 BC	38.18 BC	40.04 CD	3	3	2
EMS3403	*sbeIIb*-*3403*-*5*	36.70 BC	34.63 AB	29.26 AB	33.53 B	6	7	7
EMS3568	*sbeIIb*-*3568*-*6*	36.41 BC	38.45 BC	37.94 BC	37.6 C	7	5	4
EMS5890	*sbeIIb*-*5890*-*7*	39.88 C	40.15 BC	36.42 BC	38.82 CD	4	4	5
EMS3920	*ssIIa*-*3920*-*1*	26.20 A	24.20 A	24.76 A	25.06 A	10	12	8
EMS4308	*ssIIa*-*4308*-*2*	28.36 AB	25.41 A	23.27 A	25.68 A	8	10	11
Macia	control	NA	27.15 A	23.28 A	NA		8	10
Sepon82	control	24.63 A	26.05 A	22.71 A	24.46 A	11	9	12
Tx623	control	26.90 AB	24.47 A	23.94 A	25.11 A	9	11	9

Macia not evaluated in season Puerto Rico 16/17, so not part of combined analysis. Values followed by the same letter (A–E) in the same column are not significantly different

The *sbeIIb* mutants *sbeIIb*-*2703*-*1, sbeIIb*-*2773*-*2, sbeIIb*-*3194*-*3, sbeIIb*-*3218*-*4, sbeIIb*-*3403*-*5, sbeIIb*-*3568*-*6* and *sbeIIb*-*5890*-*7* produced significantly higher amylose values (> 30% amylose) compared to the *ssIIa* mutants *ssIIa*-*3920*-*1* and *ssIIa*-*4308*-*2* and the controls. In all three seasons, SbEMS 3194 had the highest amylose values. The SbEMS 2703, SbEMS 3218 and SbEMS 5890 were ranked 2nd to 4th in two environments, respectively. The *SSIIa* mutations represented by *ssIIa*-*3920*-*1* and *ssIIa*-*4308*-*2* produced similar amylose values and did not show higher amylose values compared to the controls.

### Mutations in *SBEIIb* alter amylose values and ASV phenotypes

Based on the higher amylose values of *sbeIIb*-*2703*-*1, sbeIIb*-*2773*-*2, sbeIIb*-*3194*-*3, sbeIIb*-*3218*-*4, sbeIIb*-*3403*-*5, sbeIIb*-*3568*-*6* and *sbeIIb*-*5890*-*7* allelic variants (Table 2) and sequence similarity to *ae* of maize, it was proposed that *Sobic.004G163700* could be a sorghum homolog of amylose extender. Based on this proposal, the amylose level in sorghum may depend on the number of alleles in the triploid endosperm. To test this hypothesis, each of the *sbeIIb* mutants was sterilized in A1-cytoplasm and the A-lines and B-lines of mutants and Tx623 were used in crosses to produce seeds carrying zero, one, two and three mutant alleles in the triploid endosperm. The amylose values increased in a dosage-dependent and mutant allele-dependent manner for *sbeIIb* mutants and did not increase in *ssIIa* mutants. In most cases, amylose content increased by 1% to 2% with each additional dose of *sbeIIb* in the endosperm with values greater than 30% when three mutant *sbeIIb* alleles were present (Table 3). This shift in amylose content was not observed in samples with varying doses of *ssIIa* (SbEMS 4308). Analyses of samples for ASV in 2018 showed an increase in percentage of seeds with an ASV phenotype depending on whether one, two or three mutant *sbeIIb* or *ssIIa* alleles were present in the endosperm. No ASV phenotype was observed in genotypes carrying one dose of the allele.

**Table 3:** Amylose content and ASV phenotype of grain samples from an allele dosage test in West Lafayette (WL) 2017 and 2018

Pedigree		WL2017				WL2018							
		Amylose%				Amylose%				% seeds with ASV+			
		0 dose	1 dose	2 dose	3 dose	0 dose	1 dose	2 dose	3 dose	0 dose	1 dose	2 dose	3 dose
ATx623/BTx623	Wild-type	24.79				22.80 E				0			
EMS 2703	*sbeIIb*-*2703*-*1*		26.65	28.60^c^	43.10		23.46 E	26.11^c^ CDE	36.62 A		0^b^	34^c^	100^a^
EMS 2773	*sbeIIb*-*2773*-*2*		26.93	26.94^c^	38.35		24.84 DE	25.04^c^ CDE	35.76 AB		0^b^	0^bc^	100^a^
EMS 3194	*sbeIIb*-*3194*-*3*		26.13	28.76^c^	47.29		24.72 DE	26.34 CDE	39.22 A		0^b^	8	96^a^
EMS 3218	*sbeIIb*-*3218*-*4*		26.75	28.48^c^	40.64		No seed	24.49 DE	38.28 A		No seed	38	83^a^
EMS 3403	*sbeIIb*-*3403*-*5*		No seed	No seed	34.63		25.29 CDE	21.33^c^ E	29.26 CD		0^b^	6^c^	88^a^
EMS 3568	*sbeIIb*-*3568*-*6*		26.18	25.79^c^	38.45		25.57 CDE	26.09^c^ CDE	31.06 CB		0	0^c^	100^a^
EMS 4308	*ssIIa*-*4308*-*2*		24.57	25.51^c^	25.47		24.04 E	23.28 E	25.28 CDE		0^b^	8	100^a^
EMS 5890	*sbeIIb*-*5890*-*7*		26.04	29.09^c^	39.64		24.71 DE	26.02 CDE	39.45 A		0^b^	38	100^a^

WL2018 Amylose: Values followed by the same letters horizontal and vertical are not significantly different. WL17: 2703 hand emasculated for 2 Doses. ASV tested on 32 seeds (results in %)

^a^Fewer than 32 seeds tested due to seed limitation

^b^No ASV swelling but leaching, very little surface swelling

^c^One biological replicate, all crosses were made in duplicate or triplicate but some A-lines (2 doses) did not set seeds

### The *sbeIIb* and *ssIIa* alleles show distinct paste viscosity profiles

**Fig. 5:**
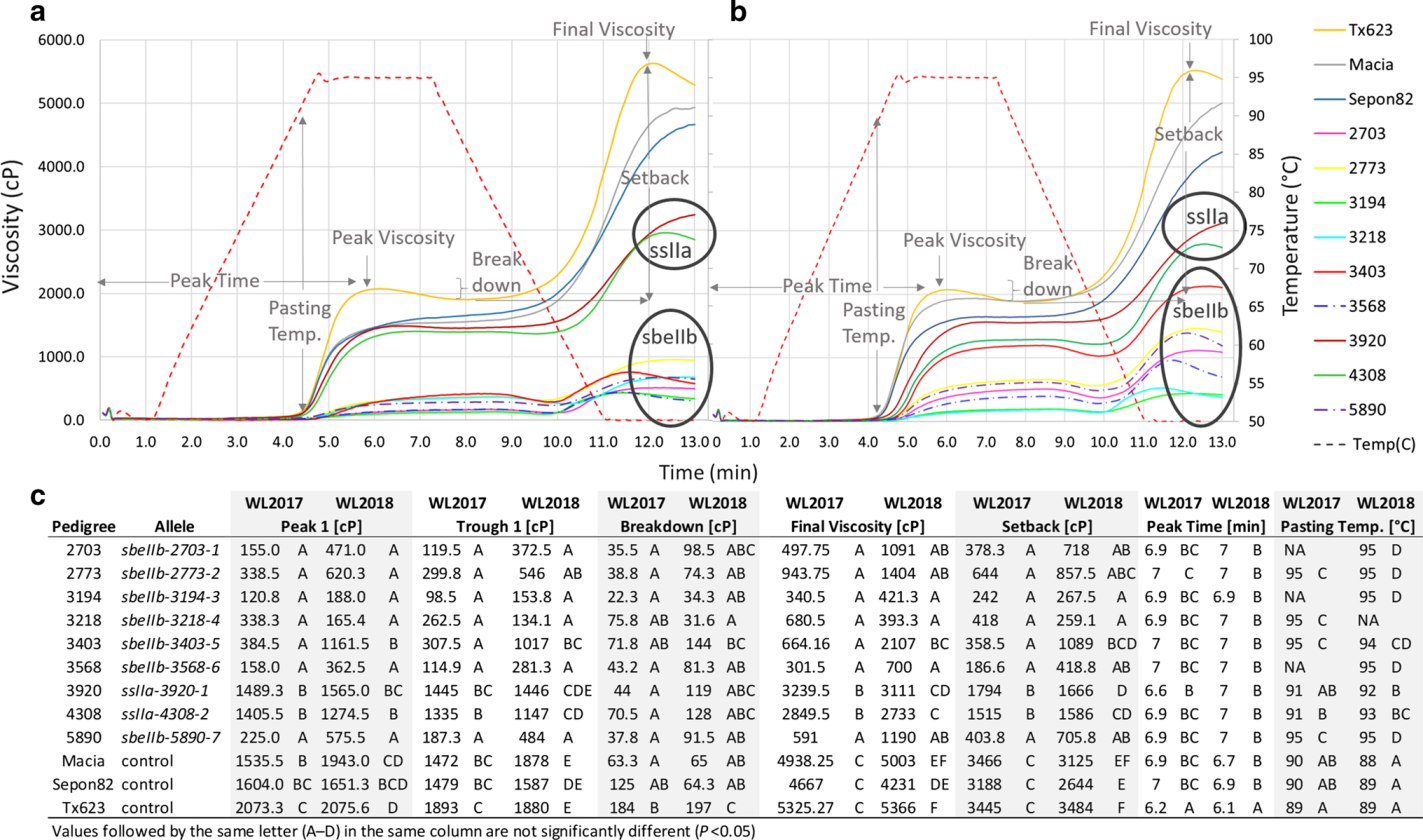
RVA paste viscosity profiles of sorghum EMS mutants and controls–lsmean values presented from two seasons **a** West Lafayette (WL) 2017 and **b** 2018 and **c** both seasons. Two biological replicates per genotype and three for BTx623 in WL2018

Pasting viscosity is an important property of starch related to its gelatinization. The paste viscosity profiles are very distinct for *ssIIa* and *sbeIIb* mutants in both environments. There was a significant genotype by environment effect for the pasting viscosity profiles (Table S2); therefore, data are presented by individual environment (Fig. 5). The *sbeIIb* mutants exhibited lower peak and final viscosity compared to *ssIIa*-*4308*-*2, ssIIa*-*3920*-*1* and controls (Fig. 5a, b). The viscosity profiles of BTx623, Sepon82, Macia, *ssIIa*-*3920*-*1* and *ssIIa*-*4308*-*2* followed the same trend in both environments. The *sbeIIb* mutants slightly overlap but cluster together across environments. The SNP mutations in *Sobic.010G093400*, encoding SSIIa, resulted in reduced viscosity in comparison with the controls but higher viscosity in comparison with *sbeIIb* mutants. BTx623 showed an earlier and larger breakdown than all other starches (Fig. 5a–c). The mutant starches demonstrated minimal breakdown and later peak viscosity or a plateau from peak viscosity. The setback viscosity, formation of a gel as the long chain amylose molecules begin to realign, was ~ 10 × lower in the *sbeIIb* mutants compared to wild-type starches.

### Double mutants of *SSIIa* and *SBEIIb* resemble the *SBEIIb* parent in phenotype

**Fig. 6:**
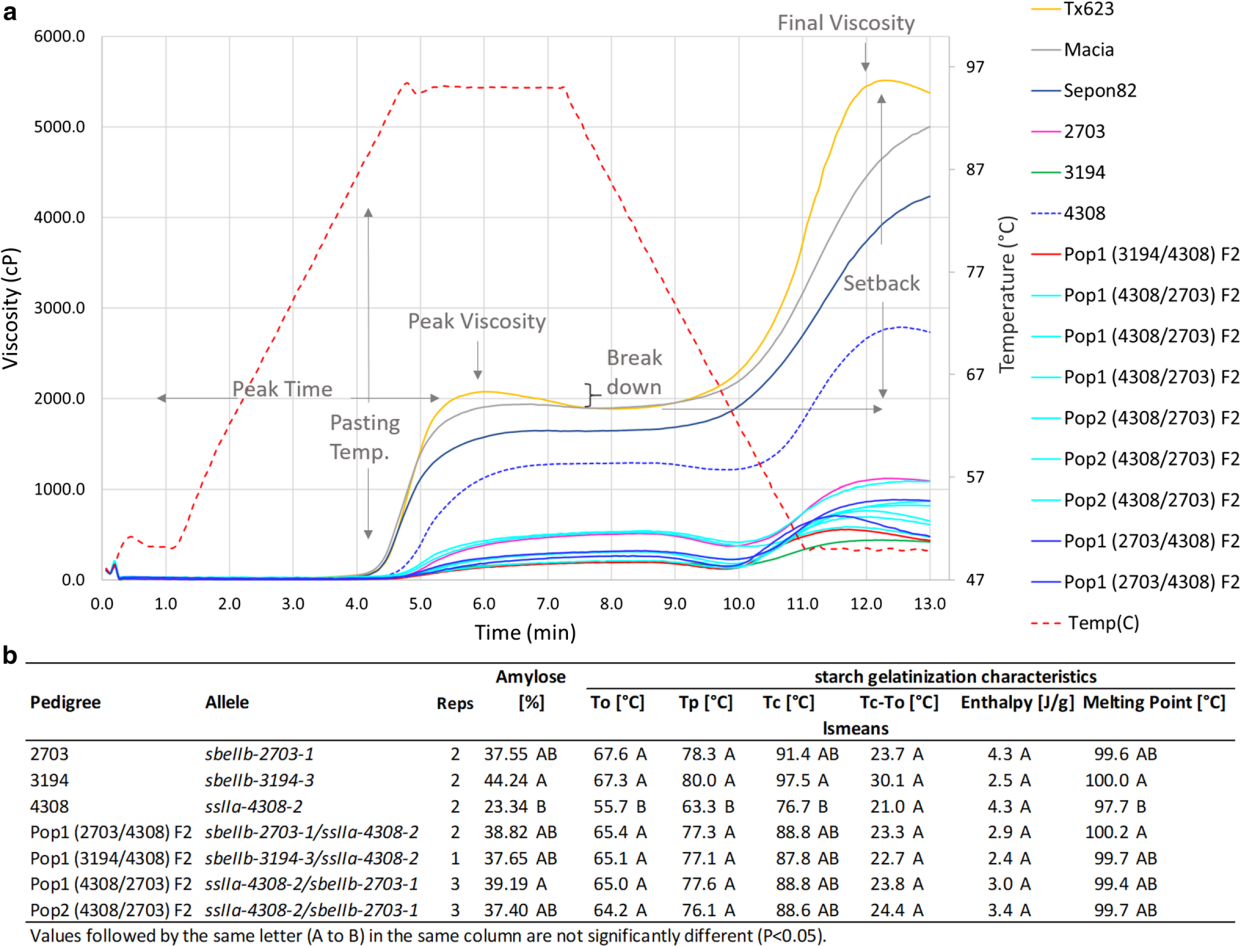
Double mutants of *sbeIIb* and *ssIIa*—**a** RVA paste viscosity profiles **b** starch thermal properties and amylose content. Pop = *F*_2_ Population, numbers (Pop1, Pop2) indicate different populations. Reps = No. of replications)

SbEMS mutant B-lines were hand emasculated and intercrossed to develop the *F*_2_ populations *sbeIIb*-*3194*-*3* × *ssIIa*-*4308*-*2* and *sbeIIb*-*2703*-*1* × *ssIIa*-*4308*-*2* (Fig. 6). Homozygous double mutants were identified in each population. Each double mutant has one parent with the *ssIIa*-*4308*-*2* allele and a second parent with either the *sbeIIb*-*2703*-*1* or *sbeIIb*-*3194*-*3* alleles. The lowest amylose values were measured in the parent carrying *ssIIa*-*4308*-*2* with 23% and higher amylose values in parent *sbeIIb*-*2703*-*1* with 38% and *sbeIIb*-*3194*-*3* with 44%. The amylose values in the double mutants resembled the *sbeIIb* mutant parent and ranged from 37 to 39%. The lowest starch GT values were measured in the parent carrying *ssIIa*-*4308*-*2* and the higher values in parent *sbeIIb*-*2703*-*1* and *sbeIIb*-*3194*-*3*. The starch thermal properties of the double mutants also resembled the *sbeIIb* mutant parent. The paste viscosity profiles varied depending on the mutant alleles involved and resulted in higher peak and final viscosity in *ssIIa*-*4308*-*2* than in *sbeIIb*-*2703*-*1* or *sbeIIb*-*3194*-*3*. The viscosity profiles for the double mutants were similar to their *sbeIIb* mutant parents.

## Discussion

Whole-genome re-sequencing of ASV mutants in sorghum revealed SNPs in starch biosynthesis genes. Seven new alleles of a starch branching enzyme (*Sobic.004G163700*) and two new alleles of a starch synthase (*Sobic.010G093400*) were identified in this study. Comparative genomics provided evidence that *Sobic.010G93400* encodes a *SSIIa*. *Sobic*.*010G093400* is most similar to SSSII-3 in rice and SSIIa in sorghum and other crop species. Sequence comparisons suggest that *Sobic.004G163700* codes for a SBEIIb with high sequence similarity to the one reported previously by Mutisya et al. (2003) and is closely related to amylose extender (SBEIIb) in maize (Fig. 2**)**. The SBEIIb of sorghum reported by Mutisya et al. (2003) showed high sequence similarity with other cereals too. The protein sequence obtained from NCBI of maize amylose extender and sorghum *SBEIIb*, AY304540, were BLAST against sorghum in the database *Phytozome* (Goodstein et al. 2012). The BLAST search exhibited the best hit for *Sobic.010G273800*, followed by *Sobic.003G213800* and *Sobic.004G163700*. All three genes are reported as similar to branching enzymes (Campbell et al. 2016), while *Sobic.003G213800* is listed as a putative 1-4-alpha glucan branching enzyme, *Sobic.010G273800* as similar to a starch branching enzyme I precursor, and *Sobic.004G163700* is similar to a 1-4-alpha glucan branching enzyme 2 (Campbell et al. 2016). No SNPs were found in *Sobic.010G273800* or *Sobic.003G213800* of ASV mutants. The gene *Sobic.004G163700* appears to be a sorghum homolog of the maize gene *amylose extender1* (*ae1*) encoding a SBEIIb.

The seven new alleles of *SBEIIb* represent moderate-to-high-impact SNP mutations spread across the entire gene and may provide a resource for understanding the gene function in sorghum. The central catalytic A-domain of SBEIIb in maize and rice has four highly conserved regions important for the catalytic activity with key amino acid positions and a catalytic triad (Tetlow and Emes 2014). The mutant allele *sbeIIb*-*3194*-*3* results in the substitution R449C, which is within a catalytic group in SBEIIb of sorghum (Mutisya et al. 2003) and the position Arg445 in maize SBEIIb (Tetlow and Emes 2014; Fig. 2). The amino acid changes in alleles *sbeIIb*-*3403*-*5* (M531I) and *sbeIIb*-*3218*-*4* (W548*) might be also in the catalytic domain of SBEIIb, as this is close to a region of importance for catalytic activity in maize SBEIIb (Tetlow and Emes 2014).

None of the nine ASV mutants with new allelic variants of *SSIIa* and *SBEIIb* have a SNP mutation in the *GBSS* (*Sobic.002G116000, Sobic.010G022600*; Campbell et al. 2016), known to act on amylose (Nakamura 2015a). It was therefore concluded that the mutants predominantly affect amylopectin synthesis. The endosperm-specific enzyme, SBEIIb, is reported to transfer shorter chains with a degree of polymerization (DP) of ~ 6 to 7 in corn, rice and wheat (Tetlow and Emes 2014; Nakamura 2015a). It is possible that the seven *sbeIIb* mutant alleles lack the enzyme function or show reduced activity, resulting in amylopectins with less short glucan chains of DP < 8 and a less branched molecule. An increase in chains of DP ≥ 12–16 in amylopectin is associated with increased starch GT (Nakamura 2015b). Therefore, having a lack of short chains might change the ratios within the amylopectin molecule and more intermediate sized chains are present, which may explain the high starch GTs of our *sbeIIb* mutants. In monocots, SSIIa is involved in the elongation of short chains (DP < 12) into intermediate size chains (DP 12–24) (Tetlow et al. 2004; Nakamura 2015b), explaining why *ssII* mutants in other species result in amylopectin with less intermediate chains and more short chains (Nakamura 2015a). An increase in amylopectin short chains of DP < 12 and a decrease in chains with DP 13 to 24 decrease starch GT (Nakamura 2015b), which could explain the low starch GT of the *ssIIa* mutants reported in this study. The *sbeIIb* mutants reported here might behave like amylose extenders with longer internal branch chains of amylopectin (Jane 2009; Tetlow and Emes 2014; Nishi et al. 2001), less branched in outer chains (Jane 2009; Tetlow and Emes 2014) and have less short chains (Jane 2009; Tetlow and Emes 2014; Nishi et al. 2001).

The ASV in sorghum is not solely controlled by a SSII as reported in previous studies in sorghum and rice (Gao et al. 2011; McKneight 2015; Wang et al. 2007) but instead is controlled by at least two genes, *Sobic.004G163700 and Sobic.010G093400*. Linkage analysis demonstrated that SNP markers for both genes co-segregated with the ASV mutant phenotype in bi-parental populations. It is possible to conclude that each SNP is either the causal mutation or linked to a gene-causing ASV that is in a larger chromosome block. The co-segregation of seven *sbeIIb* alleles in *Sobic.004G163700* with the ASV phenotype provides strong evidence that this gene is one of the genes controlling ASV in sorghum. Similarly, *ssIIa* also co-segregated with the ASV phenotype in one population supporting the conclusion that *Sobic.010G093400* also controls ASV.

The *ssIIa* and *sbeIIb* mutations result in different starch GT patterns. A comparative genomics approach showed that sorghum SSIIa is related to rice SSSII-3. The *SSSII*-*3 (ALK*) gene is the major gene that regulates starch GT (starch GT measured by ASV) in rice (Tian et al. 2009). The proposed *ssIIa* alleles in sorghum reported in the current study lower starch GT by ~ 10 °C, similar to maize (Zhang et al. 2004; Liu et al. 2012b), while a study of natural variation in *ssIIa* in sorghum (Hill et al. 2012) showed no such distinct changes in starch GT. The lower starch GT is reported to be related to amylopectin structure of *ssII* mutants (Preiss 2009). The *sbeIIb* allelic variants behave differently and confirm earlier reports that higher amylose contents are observed together with an increase in starch GT (starch GT measured by ASV) (Tian et al. 2009). In transgenic rice, the position of mutations in the *ALK* gene influenced whether ASV scores were correlated with starch GT (Gao et al. 2011). The expression of ASV in sorghum occurs for *ssIIa* or *sbeIIb* allelic variants, and it was not observed that ASV expression changed for different alleles of a gene.

Amylose extender mutant starches are known in maize and rice (Shannon et al. 2009; Li et al. 2008; Liu et al. 2012a; Nishi et al. 2001). The current study reports that *Sobic.004G163700* (SBEIIb) is similar in sequence and function to *Ae1* in maize. The new sorghum *sbeIIb* alleles result in varying amylose content with higher values overall in comparison with the *ssIIa* mutants, which is similar to *ae* maize and rice starches (Shannon et al. 2009; Li et al. 2008; Nishi et al. 2001). The amylose levels measured were > 30% in this study while > 50% in maize (Li et al. 2008; Liu et al. 2012a). The amylose levels for the controls were similar to previous reports (Beta et al. 2000; Waniska and Rooney 2000; Sang et al. 2008). Mutations of *SBEIIb* could be used to produce high-amylose sorghum varieties. Different *ae* alleles in maize are reported to have different starch thermal properties (Shannon et al. 2009; Li et al. 2008; Liu et al. 2012a) such as observed in the current study with *sbeIIb* mutants resulting in higher *T*_p_ and conclusion starch GT (*T*_c_) and in a wider starch gelatinization range compared to the wild-type (Griebel et al. 2019). Hill et al. (2012) reported that starch GT increased with increased amylose content in sorghum. The *ssII* mutants showed higher amylose values in other cereals (Zhang et al. 2004; Liu et al. 2012b; Luo et al. 2015), and nearly no change in amylose content in rice (Luo et al. 2015). No change in amylose content was observed for *ssIIa*-*3920*-*1* and *ssIIa*-*4308*-*2* in sorghum, similar to reports in rice (Luo et al. 2015).

The performance of hybrids reported in this study showed that *sbeIIb* mutations are recessive with an allele dosage-dependent influence on the amylose content in the triploid endosperm. These observations are very similar to studies for *ae* in maize (Shannon et al. 2009; Nishi et al. 2001). The wild-type allele was not completely dominant over the mutant *sbeIIb* allele as reported for ae in other species (Shannon et al. 2009).

The *sbeIIb* and *ssIIa* mutants show distinct paste viscosity profiles. It was observed that *sbeIIb* sorghum mutants have a low gel consistency. In rice, the gene *Wx* controls amylose content and gel consistency (GC), both traits being negatively correlated (Tian et al. 2009). In rice, amylose content is also regulated by minor genes such as the *ALK (SSII*-*3)* gene, *SSIII*-*2, SSI* and *PUL* (Tian et al. 2009). Minor genes controlling GC in rice are *ISA, SBE3* (Tian et al. 2009) and the *ALK (SSII*-*3)* gene (Gao et al. 2011; Tian et al. 2009). The peak viscosity is related to the maximum swelling capacity of starch granules (BeMiller and Huber 2008; Bouvier and Campanella 2014) and is mostly determined by the amylopectin content of the starch (Bouvier and Campanella 2014). This property is associated with the thickening power of a starch (Biliaderis 2009). The swelling capacity and thickening power of the high amylose *sbeIIb* mutants in the present study are lower than in controls and *ssIIa* mutants, which imply that *sbeIIb* mutants are less suitable to be used as thickening agents. The breakdown is reported to depend on the starch structure with high amylopectin starches breaking down faster (Biliaderis 2009), as observed in the results for BTx623 but not for *ssIIa*-*3920*-*1* and *ssIIa*-*4308*-*2*. During cooling, the controls, *ssIIa*-*4308*-*2* and *ssIIa*-*3920*-*1* formed stronger gels, while the *sbeIIb* variants were more unstable and sometimes liquid dispersions and did not form a proper gel, which is likely related to the extent of molecule retrogradation and re-association influencing gel formation (BeMiller and Huber 2008; Biliaderis 2009; Bouvier and Campanella 2014). Higher amylopectin starches such as those produced by the *ssIIa* mutants and the controls, resulted in better gel consistency and gel volume development than *sbeIIb* mutants, as amylopectin and amylose-amylopectin interactions are the important drivers of these properties (Biliaderis 2009).

The ASV mutants fall into distinct classes regarding functional properties and effects on starch thermal characteristics, amylose levels and pasting behavior. The *sbeIIb* genetic variants exhibited higher *T*_p_ and *T*_c_, a wider range of starch GT (Griebel et al. 2019), higher amylose levels and lower peak and final viscosity compared to the controls. Peak viscosity was positively correlated with gel consistency and negatively correlated with starch GT as previously shown in rice (Gao et al. 2011). Low amylose content together with high gel consistency and low starch GT (Griebel et al. 2019) were observed in the sorghum *ssIIa* mutants carrying the alleles *ssIIa*-*3920*-*1* and *ssIIa*-*4308*-*2,* which are desired cooking qualities in rice (Wang et al. 2007). The *ssIIa* mutants exhibit a lower peak and final viscosity than the controls but higher peak and final viscosity than the high amylose *sbeIIb* allelic variants. Hill et al. (2012) reported an increase in amylose content by genetic variants of *ssIIa* and *sbeIIb* resulting in higher starch GT, higher final viscosity, high setback, low peak viscosity and low breakdown. A clear distinction was observed between *ssIIa* and *sbeIIb* genes on amylose content, starch GT and pasting behavior with associated changes in lower final viscosity, small breakdown, smaller setback and lower peak viscosity. These results might be due to the allelic variation represented in natural variation of sorghum as compared to genotypes derived by chemical mutagenesis.

Double mutants of amylose extender and *sugary2* (*su2*) have been reported to exhibit amylose values and starch GT more similar to the *amylose extender* parent in other crops (Shannon et al. 2009). The sorghum double mutants described in this study also resemble the *sbeIIb*-*2703*-*1* and *sbeIIb*-*3194*-*3* parents for amylose content, starch thermal properties and starch viscosity profiles. It is not clear if the *sbeIIb* influences *ssIIa* or is dominant over it. The *ALK (SSII*-3) gene in rice did not contribute strongly to changes in paste viscosity profiles as observed for the *wx* locus (Wang et al. 2007). Variants of the *wx* locus were not characterized in our study of sorghum mutants; however, it was observed that the allele *ssIIa*-*4308*-*3* did not contribute to the paste viscosity profiles of double mutants.

The branch chain length and branching frequency influence starch digestibility (Tetlow and Emes 2014). Therefore, it would be of great importance to further evaluate the newly identified alleles for their branch chain length distribution and branching frequency. Genotypes with an *ae* mutation and high levels of amylose are reported as being correlated with resistant starch content (Jane 2009; Tetlow and Emes 2014; Li et al. 2008) and are less enzyme-digestible (Tetlow and Emes 2014; Li et al. 2008). It may be valuable to determine whether the *sbeIIb* mutants produce more resistant or slowly digested starches.

Nine novel alleles of a *SBEIIb* and *SSIIa* were identified and characterized in a *Sorghum bicolor* EMS population. These mutants exhibited an ASV phenotype and varying starch functional properties. The ASV phenotype was controlled by *SSIIa* and *SBEIIb.* The *sbeIIb* variants resulted in lower paste viscosity profiles, lower gel consistency, lower thickening power, higher amylose levels and higher starch GT (especially *T*_c_) than wild-type samples or ssIIa mutants. The *ssIIa* mutant alleles resulted in a drop of final viscosity, less gel consistency, lower thickening power, same amylose values and lower starch GT compared to wild-type samples from Tx623. These mutations provide opportunities to produce sorghum varieties with modified starch end-use qualities likely important for the beer brewing and baking industries and specialty foods for humans with diabetes. The fast and cheap ASV assay adapted to sorghum can be easily implemented as a pre-selection step in plant breeding programs around the globe. However, the ASV assay will not completely replace the use of DSC and RVA technologies and requires those analyses on pre-selected ASV+ genotypes.

## Electronic supplementary material

Below is the link to the electronic supplementary material.

Supplementary material 1 (DOCX 22 kb)
