# Supplementary material for: Mutations in sorghum SBEIIb and SSIIa affect alkali spreading value, starch composition, thermal properties and flour viscosity
Source: Theor Appl Genet. 2019 Oct 17;132(12):3357–74. doi: 10.1007/s00122-019-03430-0 (PMC6820604; doi:10.1007/s00122-019-03430-0)
Supplement: Supplementary file 1 — Supplementary material 1 (DOCX 22 kb) [file 122_2019_3430_MOESM1_ESM.docx]

Supplemental tables

# Table S1: ANOVA of fixed effects of amylose data from Puerto Rico 2016/2017, West Lafayette 2017 and 2018.

|  | Type III Test of Fixed Effects | |
| --- | --- | --- |
| Effect | F | Pr > F |
| Environment | 4.72 | 0.0167 |
| Pedigree | 115.13 | <.0001 |
| Environment*Pedigree | 0.49 | 0.9506 |

# F = F-value

# Table S2: ANOVA of fixed effects of paste viscosity data from West Lafayette 2017 and 2018.

|  | | | | | | | | | | | | Type III Test of Fixed Effects | | | | | | | | | | |
| --- | --- | --- | --- | --- | --- | --- | --- | --- | --- | --- | --- | --- | --- | --- | --- | --- | --- | --- | --- | --- | --- | --- |
|  | Peak 1 |  | | Trough 1 | | | | Breakdown | |  | | Final Viscosity | | | Setback | | | Peak Time | | | Pasting Temp. | |
| Effect | F | | Pr > F | | F | Pr > F | F | | Pr > F | | F | | Pr > F | F | | Pr > F | F | | Pr > F | F | | Pr > F |
| Environment | 28.44 | | 0.0001 | | 24.49 | 0.0003 | 20.55 | | 0.0013 | | 10.5 | | 0.0071 | 2.34 | | 0.1554 | 0.28 | | 0.6072 | 0.9 | | 0.3620 |
| Pedigree | 99.70 | | <.0001 | | 106.85 | <.0001 | 8.89 | | 0.0007 | | 146.43 | | <.0001 | 146.68 | | <.0001 | 44.98 | | <.0001 | 138.65 | | <.0001 |
| Environment *Pedigree | 4.03 | | 0.0107 | | 4.17 | 0.0095 | 5.02 | | 0.0117 | | 3.66 | | 0.0191 | 4.74 | | 0.0099 | 1.5 | | 0.2470 | 5.21 | | 0.0074 |

# F = F-value
